# New Nano-Crystalline Hydroxyapatite-Polycarboxy/Sulfo Betaine Hybrid Materials: Synthesis and Characterization

**DOI:** 10.3390/molecules29050930

**Published:** 2024-02-21

**Authors:** Aránzazu Díaz-Cuenca, Kostadinka Sezanova, Rumiana Gergulova, Diana Rabadjieva, Konstans Ruseva

**Affiliations:** 1 Materials Science Institute of Seville (ICMS), Joint CSIC-University of Seville Center, 41092 Seville, Spain; 2 Institute of General and Inorganic Chemistry, Bulgarian Academy of Sciences, 1113 Sofia, Bulgaria; ksezanova@abv.bg (K.S.); rumigg@yahoo.com (R.G.); didiarab@svr.igic.bas.bg (D.R.); 3 Laboratory on Structure and Properties of Polymers, Faculty of Chemistry and Pharmacy, University of Sofia, 1 James Bourchier Blvd., 1164 Sofia, Bulgaria; ohtkr@chem.uni-sofia.bg

**Keywords:** hydroxyapatite, polycarboxybetaine, polysulfobetaine, Raman spectroscopy, IR spectroscopy

## Abstract

Hybrid materials based on calcium phosphates and synthetic polymers can potentially be used for caries protection due to their similarity to hard tissues in terms of composition, structure and a number of properties. This study is focused on the biomimetic synthesis of hybrid materials consisting of hydroxiapatite and the zwitterionic polymers polysulfobetaine (PSB) and polycarboxybetaine (PCB) using controlled media conditions with a constant pH of 8.0–8.2 and Ca/P = 1.67. The results show that pH control is a dominant factor in the crystal phase formation, so nano-crystalline hydroxyapatite with a Ca/P ratio of 1.63–1.71 was observed as the mineral phase in all the materials prepared. The final polymer content measured for the synthesized hybrid materials was 48–52%. The polymer type affects the final microstructure, and the mineral particle size is thinner and smaller in the synthesis performed using PCB than using PSB. The final intermolecular interaction of the nano-crystallized hydroxyapatite was demonstrated to be stronger with PCB than with PSB as shown by our IR and Raman spectroscopy analyses. The higher remineralization potential of the PCB-containing synthesized material was demonstrated by in vitro testing using artificial saliva.

## 1. Introduction

Dental caries is the most common chronic infectious disease, having serious emotional, clinical and economic consequences for patients. The caries process is not simply the cumulative loss of mineral components from tooth enamel and dentin, but a dynamic process consisting of alternating de- and remineralization occurring in the multi-parameter environment of the oral cavity. The balance between these two processes determines whether caries will develop or whether the initial damage to the integrity of the enamel will be contained and will not deepen further [1].

Saliva is a natural multicomponent system containing Na^+^, K^+^, Mg^2+^, Ca^2+^, Cl^−^, HCO_3_^−^, SCN- and H_n_PO_4_^3−n^ (n = 1, 2) ions, immunoglobulins, proteins, enzymes, mucins, urea and ammonia [2]. Lactic and acetic acids produced by the bacterial decomposition of fermented sugars are also present in saliva and can lower the pH to below 5.5 when the process of enamel dissolution or demineralization begins. The reverse process of remineralization starts when conditions are created for the crystallization of hydroxyapatite (HA), a major component of tooth enamel, and its deposition at the demineralized site. The precipitation of calcium phosphate phases from saliva is usually not observed due to the presence of salivary organic components that bind to calcium and phosphate ions in complexes, thus preventing the formation of the ion cluster and growth to the critical size required for the precipitation of a crystalline phase [3]. The effective remineralization of enamel can proceed when adequate levels of free calcium and phosphate ions are available. The development of biomaterials capable of providing calcium, phosphate and fluoride ions available at non-cavitated caries sites to promote a controlled remineralization process has been proposed as a very promising strategy for both prevention and non-invasive therapies for the treatment of caries [1,2,3,4]. Based on the solubility of fluorapatite, which is much lower than that of hydroxyapatite, fluoride-containing materials were developed for the first time to activate the remineralization process [5]. In recent years, high levels of fluoride have been found to be toxic and efforts are being directed towards the development of non-fluoride materials [6]. 

A number of unique non-fluoride calcium phosphate-based remineralization systems have been developed. Amorphous calcium phosphate [7,8,9,10], nanosized HA [11], α- and β-tricalcium phosphate [12] are used as the ion source. Their combination with different organic agents such as casein phosphopeptide [7], chitosan and its derivatives [8], polyelectrolytes [9,10], sodium lauryl sulfate [12], etc., prevents spontaneous crystallization and the formation of differently oriented disordered crystals. Silica-based bioactive glasses are another class of materials that have shown properties to stimulate HA deposition and the enamel remineralization process [13,14,15]. Their action is associated with an ion exchange between Na^+^ ions from the glasses and H^+^ ions from the saliva, which increases the pH of the medium and helps to form a silica-rich layer on which apatite crystals are deposited. 

Hybrid materials based on calcium phosphates and polymers are undoubtedly the ones that most closely resemble the overall composition and microstructure of the extracellular matrix of natural hard tissues [16,17,18] and are recommended for the remineralization of primary tooth enamel deficiencies because they facilitate quick and noninvasive enamel restoration [19]. In tooth enamel, mineralization is thought to begin with the nucleation of amorphous calcium phosphate (ACP) in an amelogenin-rich extracellular protein matrix, and amyloid-like amelogenin nanoribbons have shown to provide potent scaffolds for ACP mineralization by presenting energetically and stereochemically favorable templates of calcium phosphate ion binding [20]. The cooperative effect of interactions between proteins and inorganic ions in biomineralization is being extensively investigated. Thus, it has been reported that the particular charge distribution in terms of concentration and distribution along the biomacromolecule may affect the orientation, polymorph and morphology of the biomineral [21,22]. 

In this vein, an interesting strategy for the preparation of hybrid biomaterials that mimic the structures of natural hard tissues is the search of synthesis routes with the use of synthetic polymers. Synthetic polymers are easier to prepare due to their flexible chemistry and can be formulated to have functional groups identical to those found in dental tissue proteins, such as amelogenin, ameloblastin and enamelin, which are involved in enamel and dentin formation [23]. Fletcher et al. [24] prepared poly(vinyl pyrrolidone)–amorphous calcium phosphate hybrid materials and proved their ability to stimulate the in vitro remineralization of enamel in the presence of artificial saliva. Bonchev et al. [25] used poly(N,N′-dimethylamino ethylmethacrylate/Carbomer 940 microgels as a matrix for the deposition of calcium phosphate. These hybrid microgels were applied successfully for the remineralization of artificial caries lesions.

Because of their distinctive molecular architectures, zwitterionic polymers have garnered interest in recent years [22]. Positively and negatively charged functional groups coexist in the same repeating unit, giving the zwitterionic polymer unique properties and providing opportunities for their application as biomaterials for dentistry [26]. The high hydrophilicity of these polymers promotes the formation of a dense hydrated layer on the material surface and makes them appropriate for use as antifouling coatings [27]. In addition, pH responsiveness allows for the controlled release of medication [28]. Based on the aforementioned properties of zwitterionic polymers, we believe that it is very interesting to study their combination with calcium phosphate compounds to obtain hybrid biomaterials capable of releasing functional calcium and phosphate ions for enamel remineralization. 

This study is focused to investigate the synthesis of calcium phosphate hybrid materials using two different zwitterionic polymers, polysulfobetaine (PSB) and polycarboxybetaine (PCB), which differ in that one contains the negatively charged, sulfo functional group and one contains the carboxy functional group, respectively. We compare the effect of these two different functional groups on the formation and characteristics of the inorganic phase, as well as on the final properties of the resulting hybrids for the release of Ca^2+^ ions. Our starting hypothesis is that by using a biomimetic approach consisting of mixing the polymers with the precursor salts in a Ca/P ratio of 1.67, using an artificial physiological ionic solution and controlling the pH to values compatible with the stability of the natural hydroxyapatite phases, hybrid materials composed of a mineral phase very similar to that occurring in natural hard tissues can be obtained. The obtained hybrid materials have been characterized in order to determine their chemical composition, morphology and microstructure in comparison with a polymer-free synthesized control material. In addition, the release of Ca^2+^ ions in an artificial saliva solution has been analyzed to assess its potential functionality in promoting enamel remineralization. 

## 2. Results

### 2.1. Synthesis: Effect of the Incorporation of the Polymers in the Synthesis Medium

**Figure 1:**
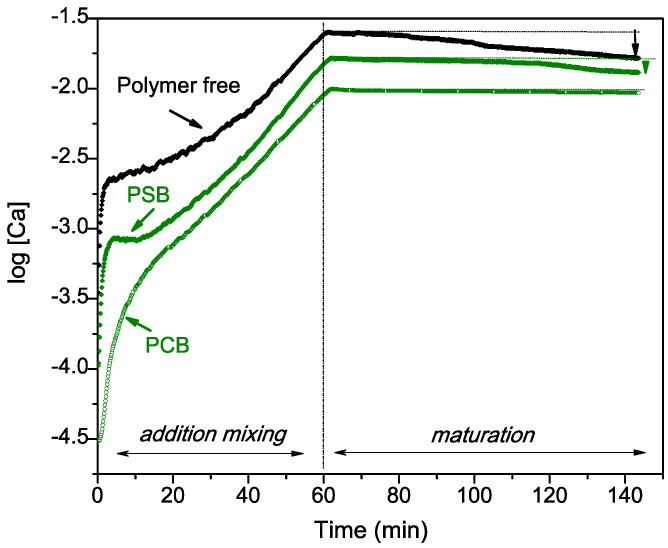
Ionic calcium concentration [Ca] profiles of the two-stage preparation, the addition and maturation of the calcium precursor solution at a constant rate for the three different types of preparations: polymer-free preparations and those synthesized using PSB and PCB.

The free ionic calcium concentration [Ca] (logarithmic scale) as a function of the synthesis time profiles for the three different types of preparations (polymer-free preparations and preparations synthesized using PCB and PSB) are displayed in Figure 1. As expected, the results show an increase in the concentration of [Ca] during the first stage of synthesis corresponding to the additive mixing of the calcium precursor solution at a constant rate. After 60 min, the addition of the Ca precursor solution is stopped, and the second stage of 80 min maturation begins. The curve profiles change to a plateau for the three preparations. This plateau is maintained with an almost constant value until the end of the maturation time for the PCB preparation. In contrast, [Ca] decreases by 12% for the PSB-synthesized preparation and by 32% for the polymer-free preparation, which shows the biggest [Ca] decrease. During the two-stage process, the addition and maturation of the calcium precursor solution, the highest [Ca] was recorded for the system without a polymer. Precipitation and dissolution processes may continue to occur simultaneously during the maturation process as the systems have not reached their equilibrium state. The presence of a polymer in the system may reduce the amount of free Ca^2+^ whilst interfering with the new formation of HA. 

### 2.2. Materials Characterization: Chemical Composition, Microstructure and Morphology

Our chemical analyses of the obtained materials are presented in Table 1. Ca/P molar ratios close to 1.67 are characteristic of the calcium phosphate apatite crystal phases [29]. Values below 1.67 for the polymer-free preparations indicate a calcium deficiency. Values higher than 1.71 for the material obtained using PCB are characteristic for B-type carbonate apatite [30].

**Figure 2:**
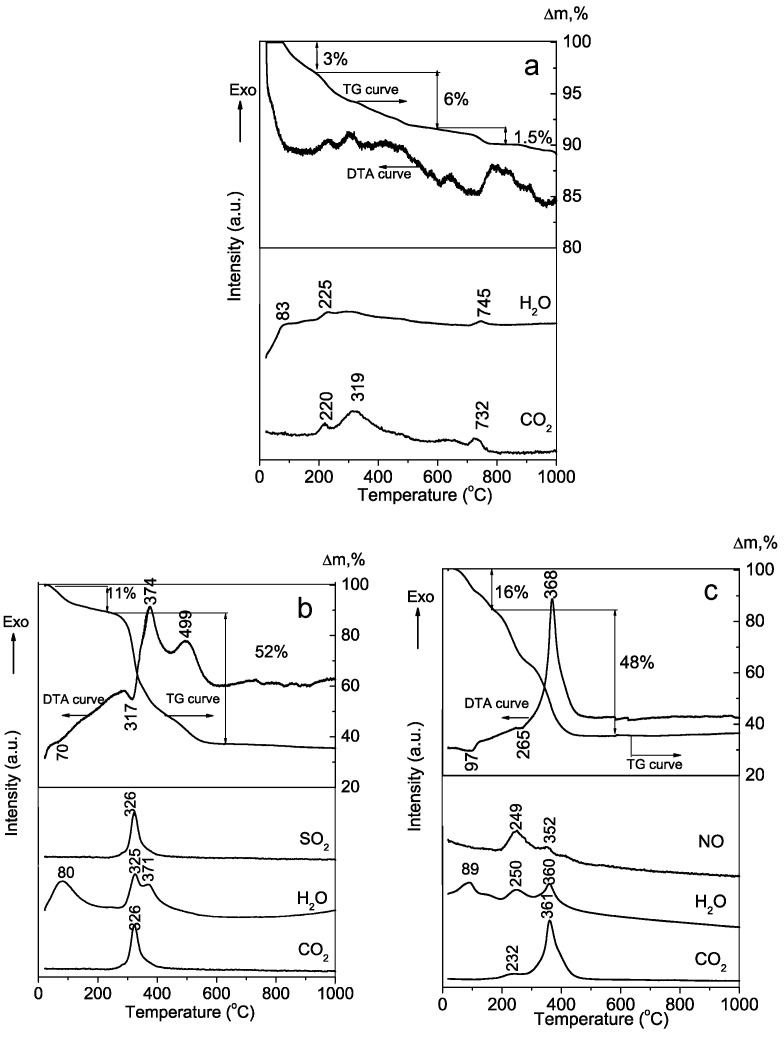
Differential thermal thermogravimetry/mass spectrometry analysis of the calcium phosphate synthesized materials: (**a**) Polymer-free; (**b**) PSB; and (**c**) PCB systems.

The polymer content in the as-prepared hybrid materials was determined by a differential thermal thermogravimetric/mass spectrometry (DT-TG-MS) analysis (Figure 2). As can be seen in Figure 2 (b and c plots), the polymers burn out within the 200–600 °C temperature range, as is evidenced by the strong exothermic effect displayed in the DTA profiles registered for the hybrid materials. These exothermic peaks correlate with the simultaneous release of CO_2_, H_2_O, and SO_2_ or NO, respectively, for the PSB and PCB systems as detected by the MS analysis, as well as a sharp weight loss of 52 and 48%, respectively, for the PSB and PCB hybrids. The DTA-TG-MS analysis of the polymer-free synthesized material (Figure 2a) is typical for nano-crystalline HA [31], which is characterized by very minor effects over the entire study and a weight loss of 10.5%. As expected, in contrast to the hybrid materials, the weight loss in the 200–600 °C interval is much lower, with only a 6% loss corresponding to the absorbed CO_2_ and chemically bonded water release. From this weight loss of 6% for the polymer-free material, not including the losses recorded for the hybrid materials in this temperature interval, the polymer content is estimated at 46% and 42% for PSB and PCB hybrids, respectively.

**Figure 3:**
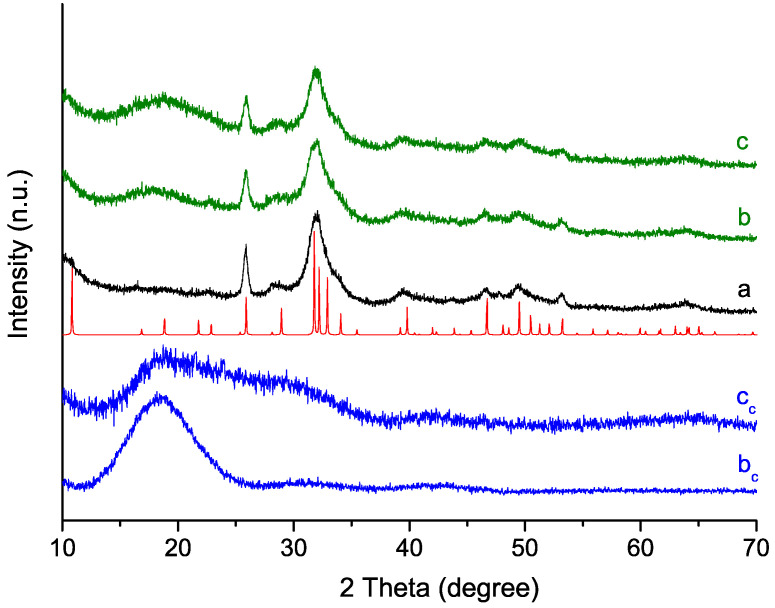
X-ray diffraction analysis of the synthesized materials: (a) Polymer-free; (b) PSB hybrid; (c) PCB hybrid. (b_c_) PSB and (c_c_) PCB pure polymer control materials. Red line peaks correspond to calcium hydroxyapatite phase (Ca_5_(PO_4_)_3_OH; ICSD collection code 60521).

Figure 3 shows the X-ray diffraction (XRD) patterns of the studied materials. The analysis indicates the formation of a crystalline phase that matched with calcium hydroxyapatite (HA; Ca_5_(PO_4_)_3_OH; ICSD collection code 60521) as a single crystalline phase. In all three cases, the low intensity and width of the peaks indicate either low levels of crystallinity [32] or nano-sized crystals [33,34]. However, the hybrid materials show a marked amorphous halo in the 2 theta degree range below 25. This halo can be seen in the patterns of the polymer control materials (Figure 3).

**Figure 4:**
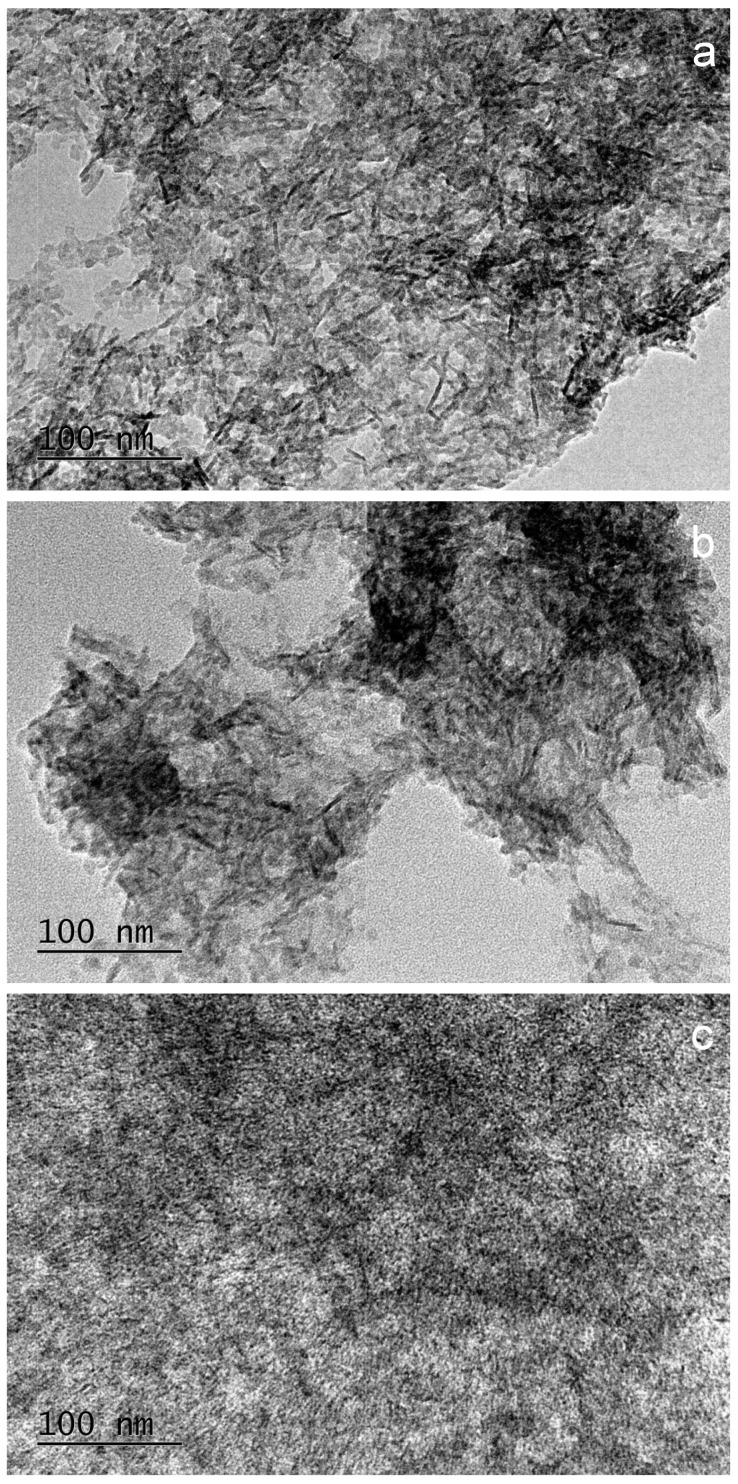
Transmission electron micrographs of the synthesized materials: (**a**) Polymer-free; (**b**) PSB; and (**c**) PCB systems.

Transmission electron microscopy (TEM) observations of the synthesized materials showed important differences in their microstructure. Their representative micrographs are displayed in Figure 4. Needle-like features up to 50 nm in length are homogeneously distributed throughout the observation area of the polymer-free material (Figure 4a). Similar needle-like nanoparticles can be observed in the PSB hybrid material, although in this case, their distribution is less homogeneous (Figure 4b). The PCB hybrid material is the one with the most different microstructure, with smaller and finer features (Figure 4c).

### 2.3. Material Characterization: Infrared and Raman Spectroscopic Analyses

**Figure 5:**
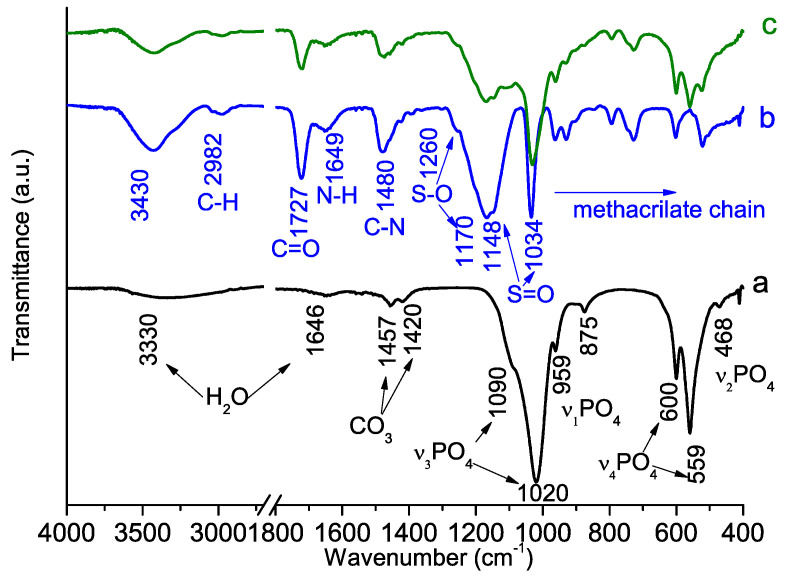
Fourier transform infrared (FT-IR) spectra of synthesized HA-PSB hybrid material: (a) HA polymer-free control material; (b) PSB polymer control material; (c) Hybrid HA-PSB material.

**Figure 6:**
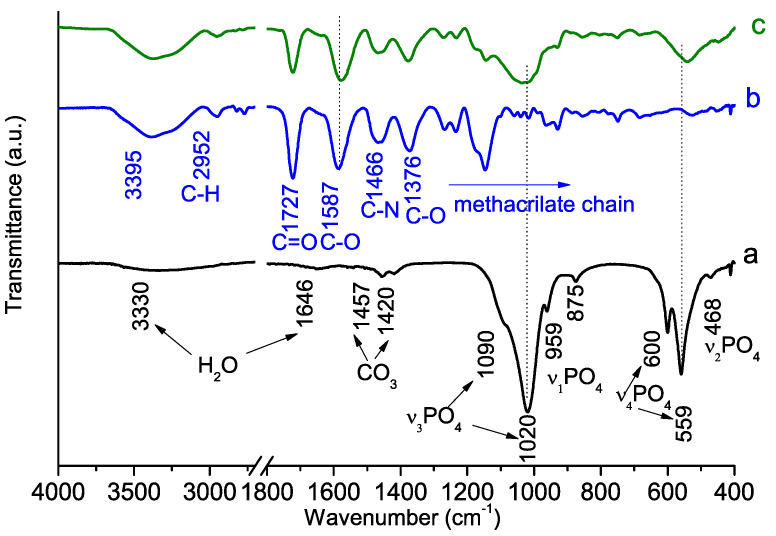
Fourier transform infrared (FT-IR) spectra of synthesized HA-PCB hybrid material: (a) HA polymer-free control material; (b) PCB polymer control material; (c) Hybrid HA-PCB material.

The infrared spectra of the synthesized hybrids and control materials (the PSB and PCB pure polymers and the polymer-free synthesized materials) are presented in Figure 5 and Figure 6. The spectrum of the calcium phosphate obtained using a polymer-free system shows characteristic vibration modes for PO_4_^3−^ at 468, 559, 600, 959, 1020 and 1090 cm^−1^ (Figure 5a and Figure 6a), which are typical for calcium hydroxyapatite (HA). The peak at 875 cm^−1^ can be attributed both to the P-OH bond characterizing calcium-deficient HA and to the CO_3_^2−^ group [35,36]. Additional bands derived from the CO_3_^2−^ group are observed at 1420 and 1457 cm^−1^. The peak with a maximum at 1646 cm^−1^ and the wide band in the 3600–3000 cm^−1^ range are ascribed to the adsorbed water molecules. The absence of well-defined peaks at 630 cm^−1^ and 3500 cm^−1^, which are characteristic of the hydroxyapatite hydroxyl group, is an indication of the non-stoichiometric nature of the obtained HA. 

The spectra of pure PCB (Figure 5b) and PSB (Figure 6b) have all the peaks characteristic of methacrylate polymers [37,38]; the bands at 3000–2700 cm^−1^ and 1300–900 cm^−1^ originate mainly from the hydrocarbon chain. The bands at 1480 cm^−1^ (Figure 5b) and 1466 cm^−1^ (Figure 6b) correspond to the C-N bonds, and the band at 1727 cm^−1^ corresponds to the carboxyl C=O groups. The main differences between the spectra of the two polymers are the strong peak at 1587 cm^−1^ (Figure 6b), ascribed to the C-O bond [37,39] and only observed for PCB, and the detection of very intense peaks at 1260, 1170, 1148 and 1034 cm^−1^ (Figure 5b) corresponding to the S-O and S=O bonds [40,41]. 

In general, the spectra of the hybrid-synthesized materials (Figure 5c and Figure 6c) exhibit main absorption peaks corresponding to both the starting polymer precursors and nano-crystalline HA. However, it is interesting to note a few important differences between the spectra of the two hybrid materials. For example, the two peaks at 600 and 559 cm^−1^ are characteristics of phosphate in a crystalline environment [42] and are well resolved only for the PSB hybrid material. Moreover, the peak at 1587 cm^−1^ ascribed to the C-O vibration detected in PCB hybrid exhibits a slight amount of broadening and shifting with respect to the PCB pure polymer. 

**Figure 7:**
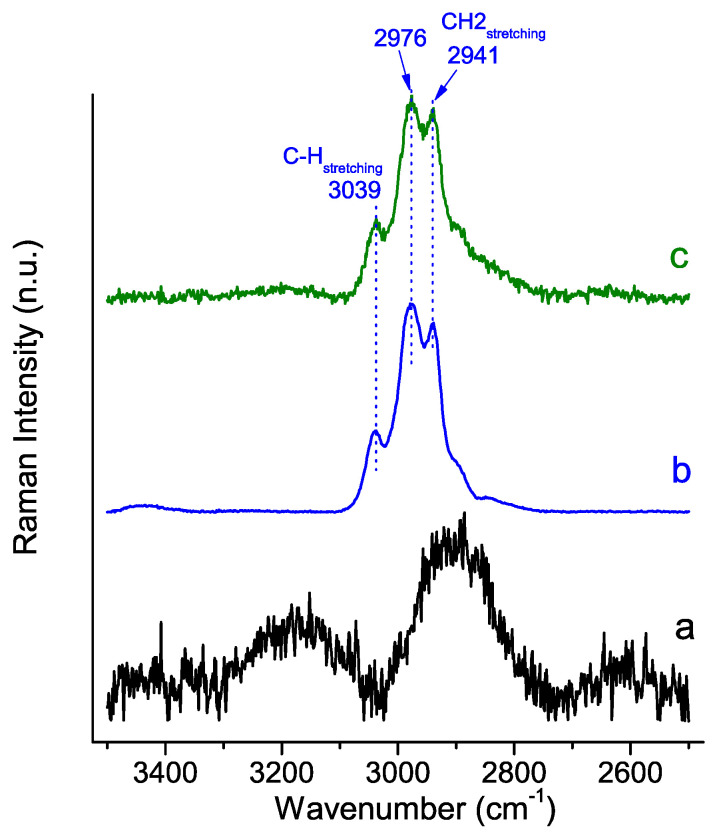

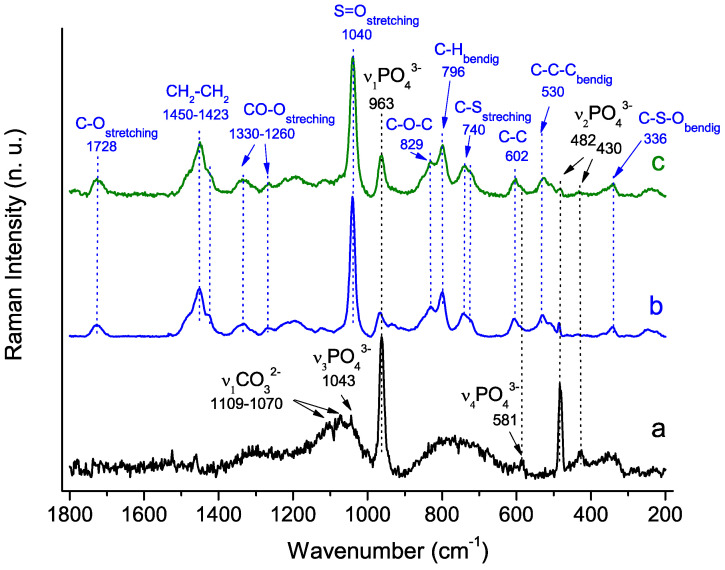
Raman analysis of synthesized HA-PSB hybrid material: (a) HA polymer-free control material; (b) PSB polymer control material; (c) Hybrid HA-PSB material.

**Figure 8:**
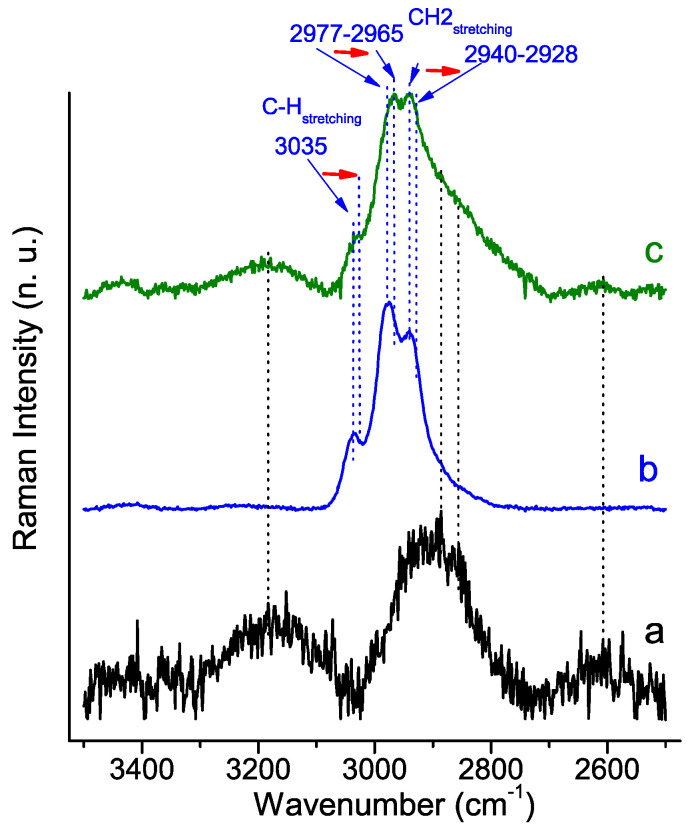

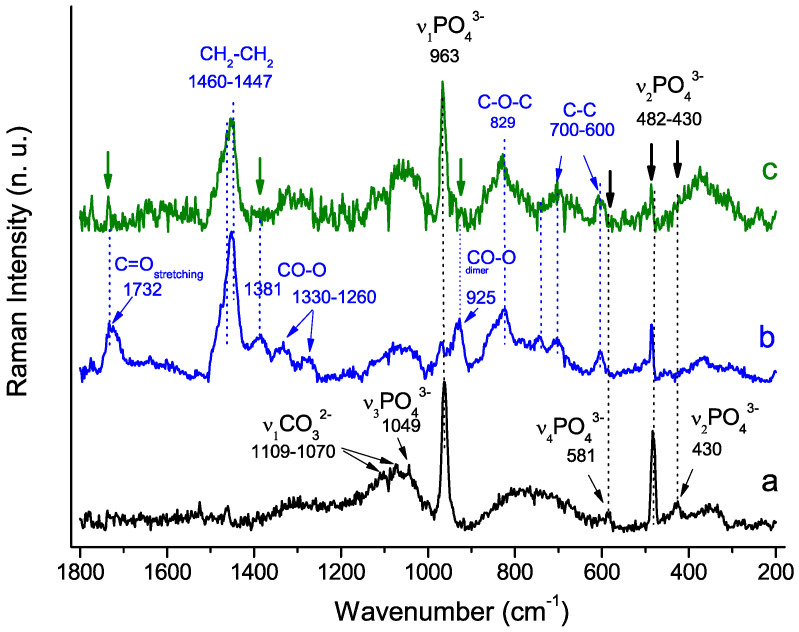
Raman analysis of synthesized HA-PCB hybrid material: (a) HA polymer-free control material; (b) PCB polymer control material; (c) Hybrid HA-PCB material.

Figure 7 and Figure 8 show the Raman spectra of the PSB and PCB hybrids, respectively, compared to those of the pure polymer control and the HA material synthesized without a polymer. The spectrum of the polymer-free system features the characteristic peaks of HA crystals [43,44], which include a very strong ν_1_PO_4_^3−^ peak at 963 cm^−1^, two ν_2_PO_4_^3−^ peaks at 482 and 340 cm^−1^, a wide band in the 1000–1200 cm^−1^ range with overlapping ν_3_PO_4_^3−^ and ν_3_CO_3_^2−^ peaks at 1049 cm^−1^ and 1109–1070 cm^−1^, respectively, and a small ν_4_PO_4_^3−^ peak at 581 cm^−1^. 

The stretching vibrations of the methylene group of the PSB and PCB polymers are observed at 3035–3039 cm^−1^ [45], 2940 and 2977 cm^−1^ [46]. Other bands common to both polymers are those that are ascribed to CH_2_-CH_2_ bonds in the range of 1450–1423 cm^−1^, CO-O ester bands at 1330 and 1260 cm^−1^, C-O-C at 829 cm^−1^ and C-C at 600 cm^−1^. On the other hand, the differences in the functional groups of the polymers are clearly evident in the Raman spectra, which show some distinctive peaks. The spectrum of PSB is characterized by the most intense peak at 1040 cm^−1^ associated with the S=O bond, as well a peaks at 740 cm^−1^ which is ascribed to the C-S stretching vibration and one at 336 cm^−1^ associated with the bending of the C-S-O bond. Also distinct in the PSB spectrum is the peak at 530 cm^−1^ that can be identified by the C-C-C bending vibrations in the terminal chain close to the sulfoxide group. On the other hand, the PCB spectrum stands out for the intensity of the peak at 1732 cm^−1^, characteristic of the carbonyl group, as well as the bands at 1381 cm^−1^ and 925 cm^−1^, which are also ascribed to vibrations of the carboxyl group [45]. 

The spectra of the synthesized hybrid materials show peaks for both the polymer and HA. However, it is the PCB hybrid that shows the most important alterations in both the peaks assigned to the vibrations associated with the polymer bonds and also to the phosphate groups of the HA. Of note is the disappearance of all peaks related to the carboxyl group (indicated by the green arrows in Figure 8). An appreciable decrease in the bending vibrations of the phosphate group is also observed. Besides, the red shift of the stretching vibrations of methylene group of PCB in the hybrid material is also significant [46]. 

### 2.4. In Vitro Test Using Artificial Saliva 

**Figure 9:**
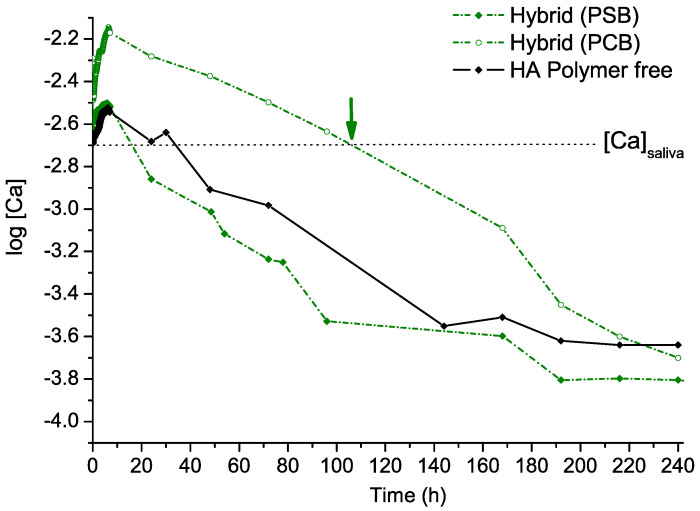
Free Ca ion time-dependent evolution profiles for the three materials synthesized in the artificial saliva fluid contact in vitro experiment.

Figure 9 shows the free [Ca^2+^] time-dependent evolution profiles for the three materials synthesized in our artificial saliva fluid contact experiment. The results show that all three materials release Ca^2+^ ions into the solutions, increasing the [Ca^2+^] above the initial value. This increase is very rapid for all three cases during the first 6 h but significantly higher for the PCB hybrid material. Furthermore, for the PCB hybrid material, this Ca concentration remains above the physiological value in saliva for a significantly longer period, over 100 h. In contrast, for the other two materials, the Ca concentration in the artificial saliva fluid decreases below the physiological value in only 24 h. It is interesting to note that artificial saliva is a metastable solution that is saturated with respect to HA. Therefore, an increase in Ca^2+^ ions will further increase this supersaturation, resulting in an increase in the thermodynamic force for HA crystallization. 

## 3. Discussion

Hybrid materials consisting of nano-crystalline hydroxyapatite as the mineral phase and polysulfo- or carboxy-betaine as the organic component were prepared using a biomimetic approach. It is well known that the wet chemistry method and the chosen variables used to produce calcium phosphate-based materials affect their phase and chemical composition. In this respect, a controlled environment and parameters as close as possible to those of the human body were used in our experiment with the aim of simulating the natural biomineralization process and producing materials with enhanced remineralization activity. The proposed biomimetic approach includes physiological solution media of NaCl of a similar concentration as that found in human blood plasma and saliva. Moreover, the PSB and PCB polymers represent organic template macromolecules, which possess simultaneously positively and negatively charged functional groups, and could thus mimic the natural building blocks of macromolecules, such as amino acids or the polar parts of phospholipids. During the synthesis process, the pH was maintained within the range of 8–8.2. We chose this pH to be close to the physiological pH (7.2–7.4) but to avoid the formation of acid calcium phosphates. In our previous study using these polymers and systems [47], but without maintaining a constant pH, we found the formation of different uncontrolled ratios of dicalcium phosphate dihydrate (DCPD), octacalcium phosphate (OCP) and even amorphous calcium phosphates (ACPs). We also proved in that previous work that the differences in the negatively charged functional groups of the two polymers influenced the calcium phosphate nucleation and the type of the final formed mineral phases, accordingly. However, under the conditions chosen in this work, we found by our XRD analysis (Figure 3) that regardless of the presence or the absence of a polymer and the polymer type used (PSB or PCB), the formation of nano-sized HA was obtained for the three different systems. This result suggests the pH control as a dominant factor in the crystal phase formation. In this respect, analyzing the changes in the concentration of free Ca^2+^ ions during synthesis could provide us with an indication of the nucleation and mineral growth processes. Hence, the difference is in the concentration of free Ca^2+^ ions, which could monitor the ability of polymers to form complexes with them. According to our results (Figure 1), the concentration of free Ca^2+^ ions in the solution is lowest in the system with PCB, followed by the one with PSB, indicating the greater ability of the carboxyl group in PCB for complexation than that of the sulfo group in PSB. This higher retention of Ca ions by the PCB also manifests itself in the maturation stage, in which the [Ca^2+^] profile only remains stable for the PCB synthesis system. At this stage, the Ca^2+^ dissolution or re-deposition processes may vary depending on the hydration properties of the two studied polymers [48]. Hence, for the polymer-free system and also to a lesser extent for the system with PSB, the systems have not reached their equilibrium, and precipitation or re-deposition predominates. In fact, the decrease in [Ca^2+^] is greatest in the polymer-free system. The presence of the polymer in the system promotes Ca^2+^ complexation in the solution, which reduces the amount of free Ca for the formation of new HA. The dipole moment of the PCB zwitterionic structure is stronger than that of PSB [49] and determines the strong interactions with Ca ions, which explains why the smallest amount of free Ca^2+^ in this system was found out of all the synthesis processes. In the polymer-free system, the complexation of calcium is the weakest and is associated only with the formation of CaHPO_4_^0^, CaH_2_PO_4_^+^, CaCl^+^, etc. [50].

The microstructure of the materials obtained by the TEM analysis is consistent with our observations in our previous paper [47], in which the ACP particles obtained in the presence of PCB are thinner and smaller in size than those obtained in the presence of PSB. In addition, and in good agreement with the observations in the previous paragraph, the large morphological difference resolved by the TEM analysis between the materials synthesized without a polymer and with PSB with respect to the PCB hybrid material indicates a greater ability to anchor Ca ions in specific positions of the PCB polymer that could act as a template to nucleate the HA mineral phase. In fact, the crystal growth may vary depending on the incipient nuclei formation, which could be dependent on the surrounding molecules such as the different polymer matrices [51]. 

The IR spectra of the newly formed materials exhibit the absorption peaks corresponding to both the initial polymers and the nano-crystalline hydroxyapatite precipitated in a synthesis run without the presence of polymers (Figure 5 and Figure 6), which confirms the existence of the mineral and organic phases in them. Significant differences in the shape and position of the peaks corresponding to the vibrations of the PO_4_^3−^ group are observed in the IR spectra of the material obtained in the presence of PCB. The broadening and rounding of the peak at 1020 and 559 cm^−1^ as well as the disappearance of the splits at 959, 600 and 468 cm^−1^ observed in the spectrum of pure calcium phosphate indicate a decrease in the crystallinity of the phosphate phase. Shifts in the peak positions of the phosphate group vibrations and the peak positions of the C–O vibrations in the carboxyl group may result from intermolecular interactions between the precipitated nano-crystalline hidroxiapatite and PCB. 

No noticeable differences in the shapes and positions of the peaks compared to those of the parent products were observed in the IR spectra of the material obtained in the presence of PSB. This is an indication of little intermolecular interaction between the two phases. 

The Raman analysis is very clear in confirming the interaction of the PCB polymer with the mineral phase. In this sense, in the PCB hybrid material, and unlike what was observed for the PSB hybrid, both the red shift of the methylene group’s stress vibrations and the elimination of the bands associated with the carboxyl groups confirm this. In the same line, the data obtained from the characterization of the HA mineral phase by XRD, TEM and FT-IR for the PCB hybrid material show a smaller HA mineral phase and less crystallinity compared to the other two materials. In this connection, it is well known that carboxylic groups containing organic compounds are known to regulate the nucleation and crystallization of hydroxyapatite in natural hard tissues [52]. 

The observed differences in the FT-IR and Raman spectral characteristics of the two hybrid materials, in relation to the corresponding precursor polymer, can be explained by the different nature of the negatively charged functional groups in the polymers, which results in a different dipole moment of the formed zwitterionic (betaine) structure. Probably, the stronger dipole caused by the shorter distance of the zwitterionic chain of PCB compared to PSB leads to stronger intermolecular interactions.

Both materials are suitable for enamel remineralization due to their capacity for the release of Ca^2+^ (Figure 9) and PO_4_^3−^ ions, which stimulate an increase in salivary supersaturation with respect to the HA phase. The supersaturation effect of the ions promotes their deposition in the form of HA at the interface with the enamel. In addition, upon the contact of the hybrid materials with the artificial saliva, simulating the environment in the oral cavity, the hydration of the polymers and the formation of hydrogels takes place. The hydrogel formed by PCB is much denser than that formed by PSB and does not allow the passage of either the Ca^2+^ ions released from the dissolution of the apatite phase or the other components of the artificial saliva, suppressing additional complex formation. The higher concentration of free Ca^2+^ ions and their longer retention in the solution make the hybrid material with PCB more promising for the remineralization of tooth enamel than that with PSB. This work can be used as a preliminary step to design further experiments on natural dental materials. More comprehensive studies are needed to evaluate the enamel remineralization capabilities and clinical outcomes of these materials.

## 4. Materials and Methods

### 4.1. Synthesis of Polymer-Free and Hybrid Materials

The synthesis of PCB and PSB by reversible addition–fragmentation chain-transfer (RAFT) polymerization has been described in detail elsewhere [47]. Briefly, PCB was prepared by dissolution of ten grams of the monomer carboxybetaine and 0.1% of 2,2-azobis (2-methylpropionamide) dihydrochloride in forty-five milliliters of acetate buffer (pH = 5.2). An appropriate amount of 0.01 mol% 4-cyano-4-(thiobenzoylthio)pentanoic acid (CTPA) calculated to obtain a linear polymer with a relative molar mass of 100,000 g mol^−1^ was neutralized with 0.05 mol dm^−3^ KOH and was added dropwise to the monomer solution. The procedure for preparation of PSB was similar. Five grams of the monomer sulfobetaine methacrylate were dissolved in twenty milliliters of acetate buffer with pH of 5.2. The initiator of 0.1 mol% 2,2-azobis(2-methylpropionamide) dihydrochloride was added. CTPA was neutralized with NaOH, and the resulting solution was added dropwise to the reaction mixture. After mixing all reagents for PCB or PSB preparation, the solutions were heated at 70 °C for 5 h and then placed in an ice bath to stop the polymerization process. The polymers were dialyzed against distilled water using a dialysis membrane (3.5 K MWCO, 16 mm, SnakeSkin™ Dialysis Tubing, Thermo Scientific, Waltham, MA, USA) until no more traces of residual monomers and other reagents were detected in the wastewater. The resulting polymer was lyophilized. The obtained PCB has an average relative molecular mass (Mr) of 75,200 with polydispersity index (PDI) of 1.2, while PSB has Mr of 98,140 and PDI of 1.429, as obtained via gel permeation chromatography.

The hybrid materials were synthesized at room temperature using a biomimetic approach as indicated as follows. A physiological solution of 0.9% NaCl was used to prepare a final solution of 0.05 mol dm^−3^ calcium and 0.03 mol dm^−3^ phosphate. NaCl (Sigma-Aldrich, St. Louis, MO, USA, A.R.), CaCl_2_·2H_2_O (Sigma-Aldrich, A.R.) and Na_2_HPO_4_ (Merck, Darmstadt, Germany, A.R.) were used. In the first step of the synthesis route, as-prepared PCB or PSB were dissolved in 180 mL calcium solution with Ca^2+^ molar ratio (monomeric unit) of 1 to 1. In the second step, the calcium solution, without polymer or with the corresponding PSB or PCB, was added to 180 mL phosphate solution at a rate of 3 mL min^−1^ by means of a combined apparatus for automatic titration and controlled synthesis (Titrando 907, Methrom AG, Herisau, Switzerland). The pH of the solution was kept constant in the 8.0–8.2 range using 0.05 mol dm^−3^ NaOH, both during the addition mixing of calcium precursor solution and subsequent maturation time. 

Concentration of free Ca^2+^ ions was monitored via Ca^2+^ polymer membrane ion selective electrodes (Methrom AG, Switzerland). The concentration of free Ca^2+^ ions was recalculated using calibration curve prepared by measurement of electrical potential of series of CaCl_2_ solutions in 0.9% NaCl. 

After 80 min maturation, the obtained suspension was transferred into a dialysis tube (3.5 K MWCO, 16 mm, SnakeSkin™ Dialysis Tubing, Thermo Scientific, Waltham, MA, USA) and washed out using distilled water, which was changed periodically until Cl^−^ ions disappeared and then finally lyophilized. 

An entirely inorganic material (named HA polymer-free control) was also prepared following an analogous synthesis route and post-treatment procedure without using polymer.

### 4.2. Characterization

#### 4.2.1. Chemical Analysis

Calcium and phosphate ions in solid phases were determined via complexometric and spectrophotometric analyses, respectively. The solids were dissolved in nitric acid. Ca^2+^ ions were determined by titration with 0.05 mol dm^−3^ EDTA at pH of 10 and indicator eriochrome black T. To determine the amounts of P (PO_4_^3−^) ions, Merck Spectroquant test kits and NOVA 60 (Merck, Germany) spectrophotometer were utilized. Ten parallel independent measurements were performed for each essay. The statistical analysis of the data was carried out using Microsoft Excel 2016. The accuracy of the results is expressed by the standard deviation value. The accuracy of the Ca/P ratio was calculated using the following equation:

$$SR=\sqrt{\frac{s_{Ca}^{2}}{C_{Ca}}}+\sqrt{\frac{s_{P}^{2}}{C_{P}}}$$

where SR—standard deviation of Ca/P molar ratio; *s_Ca_*, *s_P_*—standard deviation of Ca and P measurements; and *C_Ca_* and *C_P_*—average value of measurements of concentration of Ca and P in mmol g^−1^. 

#### 4.2.2. Powder X-ray Diffraction Analysis

Powder X-ray diffraction was performed using a Bruker (Billerica, MA, USA) D8 Advance diffractometer with CuK radiation and a LynxEye detector (Bruker AXS Advanced X-ray Solutions GmbH, Karlsruhe, Germany) in the 10 to 90° 2*θ* range with a step of 0.03° 2*θ* and a counting rate of 57 s/step. ICSD database was used for phase composition identification. 

#### 4.2.3. Differential Thermal Thermogravimetry–Mass Spectrometry Analysis (DTA-TG-MS)

LABSYS^TM^ EVO (Setaram, Caluire-et-Cuire, France) apparatus with a Pt/Pt-Rh thermocouple was used for the thermal characterization of the samples. The analysis was performed in a corundum crucible at a heating rate of 10 °C min^−1^ within 25–900 °C temperature range and in synthetic air atmosphere. A quadrupole mass spectrometer (Pfeiffer vacuum OM-NISTAR, GSD 301, Zürich, Switzerland) was part of the device and was utilized for the analysis of escaped gas. In the studied samples, the release of H_2_O (m = 18) and CO_2_ (m = 44) was determined.

#### 4.2.4. Transmission Electron Microscopy (TEM)

The hybrid materials were examined via transmission electron microscopy (JEOL transmission electron microscope, JEM-2100, Akishima City, Japan). Samples were washed several times with water and centrifuged in order to eliminate polymers and secondary crystallization from matter solution. The water-dispersed powders were then sonicated for 1 min and dropped onto standard carbon-copper grids.

#### 4.2.5. Infrared Spectroscopy (IR)

Infrared spectra were recorded using an IRAffinity-1 Fourier transform infrared (FT-IR) spectrophotometer (Shimadzu Co., Kyoto, Japan) with MIRacleTM reflection attachment (PIKE Technologies, Fitchburg, WI, USA). The vibrational spectra were obtained in the 400–4500 cm^−1^ range directly on the material as synthesized without prior sample preparation. 

#### 4.2.6. Raman Spectroscopy 

Raman spectra were collected using a dispersive Horiba Jobin Yvon (Palaiseau, France) LabRam HR800 confocal Raman microscope with a green laser (532.05 nm), working at power of 5 mV and using 600 groove/mm grating. The microscope used a 100× objective with a confocal pinhole of 1000 μm.

#### 4.2.7. In Vitro Tests Using Artificial Saliva

The experiments were performed using 0.25 g of material deposited at the bottom of a flat 60 mL container, which was immersed in 15 mL of artificial saliva solution (ASS) with composition according to Klimek et al. [53] under static conditions at room temperature. The ASSs were prepared by subsequent dissolution of 0.33 g L^−1^ KH_2_PO_4_ (Merck, Darmstatd, Germany). 0.34 g L^−1^ Na_2_HPO_4_ (Merck, Darmstatd, Germany), 1.27 g L^−1^ KCl, (INEOS, A.R., London, UK), 0.16 g L^−1^ NaSCN (Sigma-Aldrich, St. Louis, MO, USA, ACS reagent, ≥98.0%), 0.58 g L^−1^ NaCl (INEOS, A.R., London, UK), 0.17 g L^−1^ CaCl_2_ (Sigma-Aldrich, St. Louis, MO, USA, A.R), 0.16 g L^−1^ NH_4_Cl (Reanal, Budapest-Hungary), 0.2 g L^−1^ urea (Sigma-Aldrich, St. Louis, MO, USA, A.R.), 0.03 g L^−1^ glucose (Sigma-Aldrich, St. Louis, MO, USA, A.R.), 0.002 g L^−1^ ascorbic acid (Sigma-Aldrich, St. Louis, MO, USA, A.R.) and 2.7 g L^−1^ mucin from Type II porcine stomach (Sigma-Aldrich, St. Louis, MO, USA) in 1L of distilled water. pH was 6.3. Commercial mucin was purified using dissolution/liophilization process.

The change in free Ca^2+^ ion concentration with the time were measured via Ca^2+^ polymer membrane ion selective electrode (Methrom AG, Switzerland), which was placed 3 mm above the material. The concentration of free Ca^2+^ ion was recalculated using calibration curve prepared using the measurement of electrical potential of series of CaCl_2_ solutions in artificial saliva.

## 5. Conclusions

This work details a procedure for the synthesis of hybrid polymer–calcium phosphate materials with a biomimetic approach that consists of the use of two types of polymers, polycarboxy (PCB) or polysulfo betaine (PSB) as template molecules, in combination with a physiological Na^+^ and Cl^−^ ion concentration, the ratio of the precursor salts of the ceramic component of calcium phosphate Ca/P = 1.67, and the control of the pH synthesis process in a very narrow range between 8.0 and 8.2. The results indicate that this procedure achieves, in both cases, hybrid materials with a polymer percentage of around 50% and a nano-crystallized calcium hydroxyapatite phase. However, the use of PCB leads to a higher degree of interaction with the inorganic precursor salts, resulting in a material with a finer microstructure in the mineral phase. This difference between the two final hybrid materials also has consequences for the final properties, as the PCB hybrid has a higher bioactivity for remineralization in the in vitro artificial saliva test. 

## Figures and Tables

**Table 1:** Chemical analysis of the synthesized materials.

Material	Ca (mmol g^−1^)	P (mmol g^−1^)	Ca/P
Polymer-free	8.76 ± 0.03	5.38 ± 0.02	1.63 ± 0.02
Hybrid (PSB)	4.07 ± 0.01	2.43 ± 0.01	1.67 ± 0.01
Hybrid (PCB)	5.12 ± 0.02	2.99 ± 0.02	1.71 ± 0.02

## Data Availability

The data presented in this study are available on request from the authors.
